# Anti-amyloid treatment in Alzheimer disease: updated implications for patients with cerebral amyloid angiopathy and other cerebrovascular conditions

**DOI:** 10.3389/fneur.2026.1889769

**Published:** 2026-08-19

**Authors:** Matija Zupan, Polona Rus Prelog, Milica Gregorič Kramberger, Senta Frol

**Affiliations:** 1 Department of Vascular Neurology, University Medical Centre Ljubljana, Ljubljana, Slovenia; 2 Faculty of Medicine, University of Ljubljana, Ljubljana, Slovenia; 3 Centre for Clinical Psychiatry, University Psychiatric Clinic Ljubljana, Ljubljana, Slovenia; 4 Department of Neurology, University Medical Centre Ljubljana, Ljubljana, Slovenia; 5 Department of Neurobiology, Care Sciences and Society (NVS) Division of Clinical Geriatrics, Karolinska Institutet, Huddinge, Sweden

**Keywords:** anti-amyloid treatment, ARIA, cerebrovascular disease, multidisciplinary, stroke management

## Abstract

Anti-amyloid treatment (AAT) with monoclonal antibodies represents a major therapeutic advance in early Alzheimer disease (AD), but its introduction has important and underappreciated implications for vascular neurology. Because many patients with AD also have cerebral amyloid angiopathy and other cerebrovascular pathology, AAT influences stroke diagnosis, acute reperfusion therapy, and long-term antithrombotic management. Clinical trials of lecanemab and donanemab enrolled highly selected patients with minimal baseline cerebrovascular pathology, limiting generalizability to real-world populations with prevalent vascular comorbidity. Amyloid-related imaging abnormalities, particularly vasogenic edema and microhemorrhages, constitute a central safety concern and may clinically mimic acute ischemic stroke, complicating emergency decision-making and increasing the risk of inappropriate intravenous thrombolysis (IVT). Emerging reports of intracerebral hemorrhage following IVT in treated patients underscore the need for revised acute stroke pathways and support consideration of mechanical thrombectomy when otherwise indicated, although AAT-specific safety data are lacking. Beyond the hyperacute phase of stroke, concomitant use of anticoagulants or dual antiplatelet therapy raises unresolved questions regarding hemorrhagic risk, particularly in patients with atrial fibrillation or those requiring complex antithrombotic regimens. Alternative strategies, including left atrial appendage closure, may be considered in selected patients, although evidence in AAT recipients remains limited. As AAT enters routine practice, multidisciplinary collaboration, dedicated imaging protocols, and prospective safety registries will be essential to integrate disease-modifying AD treatment with safe and effective cerebrovascular care.

## Introduction

The advent of disease-modifying anti-amyloid treatment (AAT) with monoclonal antibodies, particularly lecanemab and donanemab, represents a paradigm shift in the management of early Alzheimer disease (AD) and mild cognitive impairment (MCI) due to AD. While these therapies have been primarily framed within the domain of cognitive neurology, their clinical deployment inevitably intersects with vascular neurology, given the profound overlap between Alzheimer pathology, cerebral amyloid angiopathy (CAA), cerebrovascular disease, and the frequent need for antithrombotic therapies in aging populations. The implications of AAT extend well beyond cognition, reshaping acute stroke care pathways, secondary stroke prevention strategies, and long-term cerebrovascular as well as cardiovascular risk management (1). In this paper, we seek to critically appraise AAT from a vascular neurologist’s perspective, synthesizing clinical trial data, regulatory frameworks, and emerging safety signals. Our intention is to highlight under-recognized implications for acute ischemic stroke (AIS) management, secondary stroke prevention, and antithrombotic strategies, while outlining future directions at the interface of neurodegeneration and cerebrovascular medicine.

### Randomized controlled trials of AAT

Seminal randomized controlled trials (RCTs) have laid the foundation for regulatory approval of the current AAT. The CLARITY-AD trial of lecanemab (2) enrolled patients aged 50–90 years with MCI or early AD and biomarker-confirmed amyloid pathology, demonstrating modest but statistically significant slowing of cognitive decline. Similarly, TRAILBLAZER-ALZ 2, evaluating donanemab (3), targeted early symptomatic AD with amyloid positivity and stratification by tau burden. Both trials were notable not only for their efficacy signals, but also for their stringent neuroimaging-based inclusion and exclusion criteria, particularly regarding cerebrovascular pathology. Patients with more than four cerebral microhemorrhages, any evidence of cortical superficial siderosis, prior intracerebral hemorrhage (ICH), large territorial infarcts, extensive cerebral small-vessel disease, or clotting disorders or conditions predisposing to amyloid-related imaging abnormalities (ARIA) were systematically excluded, effectively minimizing baseline CAA burden and hemorrhagic risk (2, 3). Both trials required rigorous MRI screening to minimize baseline cerebrovascular risk, particularly markers associated with CAA and ARIA risk (4).

### Regulatory approval of AAT across different regions

The populations in RCTs represent a substantially selected subset of patients with relatively preserved vascular integrity, a fact that becomes critically important when extrapolating results into real-world practice. Regulatory prescribing information underscores this concern. In the United States, the FDA labels for lecanemab (5) and donanemab (6) emphasize the need to confirm amyloid pathology, serial MRI monitoring, and heightened vigilance for ARIA. A key driver of ARIA risk associated with AAT is the ε4 variant of the apolipoprotein E (APOE) gene, with homozygous individuals showing the highest risk; APOE ε4 is also a well-known genetic risk factor for AD (3, 7). Consequently, APOE ε4 genotyping is recommended because ARIA risk differs by genotype (8). FDA labeling advises caution with thrombolytics or anticoagulants and emphasizes individualized risk–benefit assessment before their use in patients receiving AAT.

In the European Union, both lecanemab and donanemab are now authorized for adults with MCI or mild dementia due to AD who are APOE ε4 non-carriers or heterozygotes and have confirmed amyloid pathology (9, 10). Donanemab initially received a negative CHMP opinion because its anticipated benefits were considered insufficient to outweigh the risk of ARIA; after re-examination, the CHMP adopted a positive opinion and EU marketing authorization was granted on 24 September 2025 (10). A modified gradual titration regimen reduced the incidence and severity of ARIA while maintaining amyloid plaque reduction comparable to standard dosing (11).

Post-authorization experience with lecanemab in Japan (12) and appropriate-use recommendations from South Korea (13) illustrate regional emphasis on infrastructure, MRI surveillance, and hemorrhagic-risk mitigation. Australia has authorized both lecanemab and donanemab for APOE ε4 non-carriers or heterozygotes with biomarker-confirmed amyloid pathology (14, 15). Regulatory registration, however, does not ensure routine access. As of July 2026, neither therapy is subsidized through the Australian Pharmaceutical Benefits Scheme: a lecanemab listing submission was withdrawn, while the Pharmaceutical Benefits Advisory Committee did not recommend donanemab because its modest and uncertain clinical benefit was considered insufficient relative to the treatment burden, serious adverse effects, and health-system cost (16, 17). Private access entails not only drug-acquisition costs but also amyloid confirmation, APOE genotyping, baseline and serial MRI, specialist review, repeated intravenous infusions, and management of ARIA and infusion-related reactions. These requirements may compound socioeconomic and geographic inequities, particularly where PET, MRI, specialist, and infusion capacity are limited. Australian modeling found donanemab unlikely to be cost-effective at prevailing thresholds and identified drug cost, efficacy, and remoteness as important determinants (18). Thus, access is constrained by affordability and health-system capacity as well as biological eligibility.

Although regulatory policies differ, they share the objective of minimizing cerebrovascular complications associated with AAT. The variation between jurisdictions reflects ongoing uncertainty regarding hemorrhagic risk in routine clinical practice, where patients frequently have greater cerebrovascular comorbidity than those included in pivotal trials. Consequently, pretreatment neuroimaging, individualized risk stratification, and careful patient selection are fundamental components of safe AAT implementation. Genotype-restricted indications in the European Union and Australia may further limit eligibility compared with the broader US labels (5, 6, 9, 10, 14, 15).

### ARIA as an AIS mimic

ARIA should not be viewed solely as a transient, treatment-phase adverse effect. AAT mobilizes plaque amyloid-*β* toward perivascular clearance pathways and can bind vascular amyloid directly, producing vascular amyloid overload, antibody-mediated inflammation, blood–brain barrier leakage, and vessel-wall injury (19). In this sense, ARIA and CAA may be regarded as two expressions of an overlapping amyloid vasculopathy: ARIA with edema or effusion (ARIA-E) resembles inflammatory CAA/CAA-related inflammation, whereas ARIA-ARIA hemosiderin/hemorrhage (ARIA-H) shares the hemorrhagic phenotype of CAA. Pre-existing CAA may therefore be both substrate and target of the treatment-related response. Importantly, absence of lobar microbleeds or cortical superficial siderosis on baseline MRI does not exclude microscopic CAA or other small-vessel fragility, particularly in AD, and should not be interpreted as proof of normal vascular integrity. Clinically, ARIA may cause headache, encephalopathy, seizures, or focal deficits that mimic AIS (20, 21). An acute deficit during AAT should prompt MRI with diffusion-weighted, FLAIR, and susceptibility-weighted sequences when obtainable without clinically consequential delay (1). Non-contrast CT alone may not reliably distinguish AIS from ARIA. Severe or serious ARIA may warrant corticosteroids and treatment interruption or discontinuation according to agent-specific guidance, although treatment evidence remains limited (1, 9, 10).

### Reperfusion treatment in patients receiving AAT

The implications for IVT require particular caution. Case reports and post-marketing surveillance have described fatal multifocal ICHs following alteplase administration in patients previously treated with lecanemab or donanemab (8, 22–24). Against a backdrop of AAT-induced vascular fragility resembling or exacerbating CAA, systemic fibrinolysis raises substantial biological concern. Appropriate-use guidance for lecanemab advises against IVT in this population (25), although whether AAT should constitute an absolute contraindication in every patient remains uncertain pending further safety data (1).

Existing thrombolysis guidance addresses current AAT, not any lifetime exposure (1). It is driven by fatal ICH after IVT during active treatment and heightened early ARIA risk. No study defines remote AAT exposure as a permanent contraindication or establishes a safe wash-out interval. Risk after discontinuation may depend on the last infusion, previous or active ARIA, microbleed or siderosis burden, APOE ε4 status, and the antibody used; former recipients therefore require MRI-informed individual assessment rather than automatic exclusion. Current or recent exposure warrants strong caution and generally avoidance of IVT, whereas remote exposure remains an evidence gap.

Whether IVT is safer during maintenance lecanemab therapy remains unknown. Current recommendations do not distinguish between induction and maintenance treatment, instead advising against IVT in all patients receiving lecanemab until adequate safety data become available. Although the incidence of ARIA decreases over time, late ARIA and hemorrhagic events may still occur, and no clinical studies have specifically evaluated IVT safety during maintenance dosing (26). Consequently, maintenance therapy should be regarded as ongoing AAT, not as an established lower-risk phase.

Mechanical thrombectomy (MT) remains available for otherwise eligible patients with CTA-confirmed proximal large vessel occlusion (LVO) and avoids systemic fibrinolysis (1). When both IVT and MT would ordinarily be indicated, direct MT may be considered after individualized assessment, but direct-MT trials in general populations cannot establish safety in AAT recipients (27). Patients with prestroke dementia or disability were generally excluded from reperfusion RCTs; observational evidence does not justify dementia as an automatic contraindication, but dementia severity, baseline function, life expectancy, and patient values should be considered (28). Large-core trials, including SELECT2 and LASTE, demonstrated less disability with MT in selected internal carotid artery (ICA) or middle cerebral artery (MCA) M1 occlusions with Alberta Stroke Program Early CT Score (ASPECTS) commonly 3–5 or core volume ≥50 mL, although most survivors remained dependent and uncertainty persists for very large cores, ASPECTS 0–2, advanced frailty, or prestroke cognitive impairment (29, 30). Large infarct burden also heightens concern for edema, reperfusion injury, and hemorrhagic transformation; this risk should inform, rather than automatically preclude, treatment. Non-contrast CT with CTA is the usual rapid assessment; CT perfusion or MRI may refine evaluation of the core, collaterals, and penumbra without important delay, but imaging selection varied across large-core trials and no single advanced imaging paradigm is universally decisive. By contrast, DISTAL and ESCAPE-MeVO did not demonstrate an overall functional benefit of routine MT for medium or distal vessel occlusion (31, 32). Decisions should therefore be individualized by experienced stroke physicians and neurointerventionalists at comprehensive stroke centers, considering occlusion site, infarct extent, collaterals, delay, baseline cognition and function, ARIA/CAA markers, hemorrhagic risk, and patient preferences; no prospective evidence establishes MT as safer specifically during AAT.

### Anticoagulants and antiplatelets: safety on AAT

Beyond hyperacute stroke, chronic antithrombotic therapy raises unresolved risks. Lecanemab trials allowed selected antithrombotic use, donanemab stratification is limited, and available analyses show mixed ARIA-H signals with few macrohemorrhages (33–35). Expert guidance advises avoiding long-term anticoagulation during AAT when possible (36, 37). Antiplatelet monotherapy may be considered when indicated but is not risk-free; dual antiplatelet therapy (DAPT) and especially triple therapy remain largely unstudied.

Carotid revascularization requires separate consideration of symptomatic status, the expected benefit over intensive medical management, and the risks associated with the selected procedure. In patients with recently symptomatic severe carotid stenosis, carotid endarterectomy (CEA) remains the established revascularization strategy when the patient is otherwise eligible, whereas carotid angioplasty with stenting (CAS) represents an alternative for selected patients in whom surgery is less suitable; the benefit of CAS over intensive medical management alone has not been established in symptomatic disease, and its peri-procedural stroke risk is particularly relevant in older patients (38). The situation is less certain in asymptomatic carotid stenosis. In CREST-2, which comprised separate parallel comparisons rather than a direct comparison between CAS and CEA, CAS plus intensive medical management reduced the four-year incidence of the primary composite outcome compared with intensive medical management alone (2.8% vs. 6.0%; absolute risk difference, 3.2 percentage points), whereas the corresponding reduction with CEA was not statistically significant (3.7% vs. 5.3%) (39). However, CAS was associated with eight strokes or deaths during the first 44 days compared with none with medical management alone, and nine early strokes occurred with CEA compared with three under medical management; disabling strokes were uncommon across all groups (39). Thus, CREST-2 does not establish routine revascularization or the superiority of CAS over CEA in asymptomatic disease.

This distinction is particularly important in older patients with cognitive impairment who are being considered for AAT. Intensive medical management remains the foundation of care, and any additional benefit from revascularization must be weighed against frailty, life expectancy, competing cerebrovascular and cardiovascular risks, procedural complications, the need for DAPT after CAS, plaque and anatomical characteristics, and patient preferences (38, 39). AAT status alone should determine neither the indication for carotid revascularization nor the choice between CEA and CAS.

### Atrial fibrillation and prevention of thromboembolic complications in patients requiring anticoagulation

Age links AD and AF (40), and direct oral anticoagulants (DOACs) prevent cardioembolic stroke but may magnify hemorrhagic risk in an amyloid-fragile brain. With concomitant lecanemab and anticoagulation, ICH occurred in 2.4% of participants in phase 3 and 2.9% including the open-label extension, versus 0.1% with placebo (41). Reduced-dose DOACs cannot be assumed safer during AAT, and lecanemab appropriate-use recommendations exclude oral anticoagulants (25). If anticoagulation is interrupted because of ARIA or AAT, resumption should not follow a fixed interval or rest solely on radiological resolution of ARIA-E. ARIA-H may leave permanent hemosiderin, and absence of overt CAA markers does not exclude microscopic CAA or persisting vessel-wall injury. Because no prospective data define a safe restart point, decisions should balance cardioembolic protection against bleeding risk, considering the anticoagulation indication, ARIA type, severity and evolution, microbleeds, siderosis and white-matter disease, APOE ε4 status, blood pressure, time since the last AAT dose, plans for continued AAT, alternatives, life expectancy, and patient preferences. Multidisciplinary input from cognitive and vascular neurology and cardiology is advisable.

One potential strategy for patients with comorbid AF who are unsuitable for long-term anticoagulation is left atrial appendage (LAA) closure. Most evidence derives from studies of the WATCHMAN device (Boston Scientific, Marlborough, MA, United States) (42). A pooled analysis showed lower rates of hemorrhagic stroke, cardiovascular death, and nonprocedural bleeding with LAA closure than with vitamin K antagonists (43). LAA closure has also been reported as feasible in patients with CAA (44) and in a single patient receiving lecanemab (45). However, evidence specific to AAT recipients is extremely limited, and postprocedural antithrombotic requirements vary by device, approach, and patient characteristics. Any such strategy therefore requires individualized multidisciplinary assessment.

Epicardial approaches such as LARIAT and AtriClip avoid an intracardiac implant and have achieved high closure rates in observational cohorts (46–48). However, the evidence is indirect, the procedures carry bleeding and pericardial risks, and none has been systematically evaluated in AAT recipients. LAA closure should therefore be viewed as a potential individualized option—not an established strategy for enabling AAT—pending focused studies.

## Discussion

As AAT becomes increasingly routine, vascular neurologists and emergency physicians will increasingly encounter patients in whom Alzheimer pathology, cerebrovascular disease, and acute neurological presentations intersect, necessitating structured multidisciplinary management protocols. In the acute stroke setting, heightened caution is warranted when considering IVT or MT, with hyperacute MRI potentially improving diagnostic certainty, distinguishing AIS from ARIA, and informing individualized risk assessment, although prospective validation is needed. Systematic registry-based data collection capturing short- and long-term outcomes in patients receiving AAT—particularly those presenting with LVO, undergoing different MT techniques, or requiring antithrombotic therapy—will be essential to inform evidence-based practice. Parallel registries focused on patients with comorbid AF could clarify the safety of anticoagulation, the role of LAA closure, and their effects on cerebrovascular and cognitive outcomes, while prospective studies and reverse-translational preclinical models may further elucidate mechanisms underlying hemorrhagic risk. Factor XIa inhibition remains of interest after OCEANIC-STROKE, but its role in AF requiring anticoagulation and in AAT recipients remains unproven (49, 50). Close collaboration among behavioral neurologists, vascular neurologists, emergency physicians, neuroradiologists, and neurointerventionalists, supported by rigorous clinical and translational research, will be critical to integrating AAT safely into comprehensive cerebrovascular care.

ARIA is especially relevant because AD commonly coexists with CAA and other small-vessel disease. AAT may amplify hemorrhagic vulnerability in APOE ε4 carriers and patients receiving antithrombotics, while pivotal trials excluded patients at higher cerebrovascular risk. This limits generalizability and reinforces the need for vascular phenotyping rather than reliance on trial eligibility alone.

The long-term cerebrovascular consequences of AAT remain unclear. ARIA usually occurs early, but CAA is central to its pathobiology: antibody binding to vascular amyloid and redistribution of parenchymal amyloid toward perivascular pathways can trigger inflammation and injury in already vulnerable vessels (51, 52). Human neuropathology has linked ARIA to amyloid-laden leptomeningeal and penetrating vessels, microinfarcts, hemosiderin, complement activation, and vascular macrophage responses; ARIA-H may persist after ARIA-E resolves (53). Because AD frequently coexists with microscopic CAA that may be below MRI detection (54), disappearance of edema or completion of AAT cannot be assumed to normalize bleeding risk. Whether vascular integrity fully recovers, anticoagulation can be safely resumed, or maintenance AAT should stop when anticoagulation becomes necessary is unknown. Prospective imaging, biomarker, and outcome data are required.

The findings reinforce the need for a more integrated “neurovascular” model of cognitive decline. Future disease-modifying trials should move beyond amyloid reduction alone and incorporate rigorous vascular phenotyping, advanced MRI markers of small vessel disease, and hemorrhagic risk stratification. From a vascular neurology standpoint, optimizing modifiable vascular risk factors—including hypertension, diabetes, dyslipidemia, AF, sleep disorders, and sedentary lifestyle—may ultimately provide broader population-level benefit and superior safety than narrowly targeted AAT strategies alone. Successful dementia therapeutics may require combined neurodegenerative and vascular approaches (55, 56). Table 1 summarizes potential clinical considerations and their evidentiary limitations for vascular neurologists treating patients receiving AAT.

**Table 1:** Potential clinical considerations and evidentiary limitations for vascular neurologists treating patients receiving AAT.

Clinical scenario	Key vascular neurology concern	Potential considerations	Key cautions and limitations	Evidence basis
Acute focal neurological deficit in patient on AAT	ARIA-E/ARIA-H may mimic AIS	MRI with DWI, FLAIR, and SWI/T2* sequences may be considered when feasible and without clinically important delay	Non-contrast CT alone may be insufficient when ARIA is clinically plausible	Expert guidance, post-marketing reports, and pathophysiological rationale
Suspected AIS eligible for IVT	Increased risk of catastrophic ICH in amyloid-fragile vessels	Individualized assessment including exposure timing, ARIA history, MRI findings, and CAA markers; assess for MT if LVO is present	Routine IVT without assessment of AAT exposure, MRI findings, and hemorrhagic risk	Case reports and expert guidance; very limited direct evidence
LVO AIS	Establishing occlusion site, infarct extent, baseline cognition and function, and hemorrhagic risk	Rapid non-contrast CT and CTA; CT perfusion or MRI when it informs selection without important delay. MT may be considered for otherwise eligible proximal LVO, including selected large-core infarction, after case-by-case specialist assessment	Automatic treatment or exclusion based solely on dementia or core size; routine MT for medium or distal vessel occlusion; extrapolation of large-core trials to very large cores, ASPECTS 0–2, advanced frailty, or AAT-specific safety	Proximal-LVO and large-core RCTs; neutral medium/distal-vessel RCTs; observational dementia evidence; no prospective AAT-specific data
Baseline AAT eligibility assessment	Underrecognized CAA and small vessel disease increase ARIA risk	Baseline MRI assessment of microbleeds, siderosis, white matter disease, and other risk markers, together with APOE ε4 testing, consistent with agent-specific labeling	AAT initiation outside agent-specific eligibility and safety criteria	Pivotal-trial criteria, regulatory labeling, and expert guidance
ARIA-E/ARIA-H detected on MRI	Progression to symptomatic edema, seizures, encephalopathy, hemorrhage	Temporary withholding or discontinuation of AAT may be appropriate according to severity and agent-specific guidance; corticosteroids may be considered for severe ARIA	Routine rechallenge after serious or severe ARIA	Regulatory labeling and expert guidance; limited treatment evidence
Concomitant or resumed oral anticoagulation	Balancing cardioembolic prevention against ARIA-related and CAA-related hemorrhagic risk	Individualized multidisciplinary assessment incorporating the anticoagulation indication, ARIA type, severity and evolution, MRI hemorrhagic markers, APOE ε4 status, blood pressure, AAT timing or continuation, and alternatives	Fixed-interval DOAC resumption; assuming resolved ARIA-E or absent MRI markers excludes persistent small-vessel fragility; long-term anticoagulation with active ARIA or advanced CAA when avoidable	Mechanistic and pathological data, trial safety, and expert guidance; no validated restart interval or prospective AAT-specific DOAC evidence
AF requiring stroke prevention	Balancing cardioembolic prevention against hemorrhagic risk	LAA closure may be considered when long-term anticoagulation is unsuitable after multidisciplinary assessment	Presenting LAA closure as an established AAT-specific strategy; triple therapy when alternatives exist	General LAA-closure evidence plus limited CAA and AAT reports
Significant carotid stenosis in a patient receiving AAT	Establishing symptomatic status and whether revascularization adds sufficient benefit to intensive medical management; procedural and antithrombotic risks in older or frail patients	Intensive medical management for all patients. For recently symptomatic severe stenosis, CEA may be considered when otherwise indicated; CAS may be considered selectively when CEA is unsuitable and the required antiplatelet regimen is acceptable. For asymptomatic stenosis, any intervention requires individualized assessment of life expectancy, frailty, cognitive status, procedural risk, plaque and anatomical characteristics, and patient preferences	AAT status or stenosis severity alone should not determine intervention. CREST-2 should not be extrapolated to symptomatic disease or interpreted as establishing CAS superiority over CEA. Delayed benefit must be balanced against procedural risk and DAPT exposure	Guideline evidence for CEA in symptomatic stenosis; CREST-2 evidence for asymptomatic stenosis ≥70%; no prospective AAT-specific evidence
Antiplatelet therapy	Potential increase in microhemorrhage burden	When antiplatelet therapy is clinically indicated, monotherapy may be considered where appropriate	DAPT without a compelling indication or beyond the shortest appropriate duration	Limited observational evidence and expert guidance
Long-term vascular prevention	Mixed neurodegenerative–vascular pathology drives progression	Individualized optimization of hypertension, diabetes, dyslipidemia, AF, sleep apnea, and physical inactivity	Focusing exclusively on a single therapeutic target	General vascular-prevention evidence

AF, Atrial fibrillation; AIS, Acute ischemic stroke; APOE ε4, Apolipoprotein E epsilon 4; ARIA-E, Amyloid-related imaging abnormalities with edema; ARIA-H, Amyloid-related imaging abnormalities with hemorrhage; AAT, Anti-amyloid treatment; CAA, Cerebral amyloid angiopathy; CAS, Carotid angioplasty with stenting; CEA, Carotid endarterectomy; CT, Computed tomography; CTA, Computed tomography angiography; DAPT, Dual antiplatelet therapy; DWI, Diffusion-weighted imaging; FLAIR, Fluid-attenuated inversion recovery; ICH, Intracerebral hemorrhage; IVT, Intravenous thrombolysis; LAA, Left atrial appendage; LVO, Large vessel occlusion; MRI, Magnetic resonance imaging; MT, Mechanical thrombectomy; SWI, Susceptibility-weighted imaging. These considerations are not prescriptive recommendations. Their applicability is limited by the absence of prospective AAT-specific evidence, and all decisions require individualized multidisciplinary assessment of expected benefit, hemorrhagic risk, treatment timing, imaging findings, comorbidities, and patient preferences. “Evidence basis” summarizes the principal sources supporting each consideration and does not represent formal GRADE adjudication.

## Conclusion

Lecanemab and donanemab represent progress in early AD and MCI, but ARIA has direct implications for stroke diagnosis, reperfusion treatment, and hemorrhagic risk. Safe integration of AAT into cerebrovascular care requires collaboration among cognitive and vascular neurologists, neuroradiologists, neurointerventionalists, and cardiologists. Current evidence supports caution and individualized assessment but does not yet provide definitive AAT-specific algorithms for acute stroke or antithrombotic management. Multidisciplinary protocols, prospective registries, and longer-term safety data are therefore needed to balance disease-modifying treatment with cerebrovascular protection. Regulatory approval alone will not ensure population-level benefit unless treatment is supported by equitable reimbursement and adequate diagnostic, MRI-monitoring, specialist, and infusion-service capacity.

## Data Availability

The original contributions presented in the study are included in the article/supplementary material, further inquiries can be directed to the corresponding author/s.
